# Multimedia Gloss Presentation: Learners' Preference and the Effects on EFL Vocabulary Learning and Reading Comprehension

**DOI:** 10.3389/fpsyg.2020.602520

**Published:** 2021-02-04

**Authors:** Shufang Wang, Chang In Lee

**Affiliations:** Department of TESOL English Education, Pai Chai University, Daejeon, South Korea

**Keywords:** multimedia glosses, vocabulary learning, reading comprehension, COVID-19, L2 definition, L2 definition and video, L2 definition and picture, L2 definition and audio

## Abstract

Drawing on Moreno's cognitive-affective theory of learning with media, this research aims to investigate the effectiveness of different multimedia glosses on learners' vocabulary acquisition and reading comprehension in a CALL environment. A total of 160 university students who learnt English as a foreign language (EFL) in four classes participated in the study and were exposed to one of the four conditions: (a) L2 definition only, (b) L2 definition coupled with audio, (c) L2 definition plus video, and (d) L2 definition with picture. Participants were asked to read eight hypermediated reading texts. Reading comprehension as well as vocabulary acquisition were measured using a pretest–posttest design. The results show first that all these four conditions led to students' vocabulary gains. More specifically, participants who had access to L2 definition plus picture and plus videos achieved significantly higher scores than the other two groups, L2 definition coupled with audio and L2 definition only. Concerning reading comprehension, all multimedia glossing presentation led to better reading comprehension, but no differences were found among all the glossing groups. The questionnaire and interview data indicate that students preferred L2 definition coupled with video and picture glosses, followed by audio and L2 definition only. The results reflect that multimedia glossing presentation creates a different effect on students' vocabulary acquisition and reading comprehension, respectively. They also provide pedagogical implications for learning in times of COVID-19.

## Introduction

The advances in computer technology have presented ample opportunities for teachers and researchers for making use of it in the field of language learning and teaching. Due to the advantages of multimedia, such as accessibility, integration of different media, and efficiency, some English teachers, and researchers strive to develop several multimedia strategies to improve students' vocabulary learning and reading comprehension. One good example is the effective use of multimedia glosses, which can incorporate various forms of glosses (e.g., text, audio, videos, and pictures) into authentic texts, thereby making the texts more understandable for L2 learners and facilitating vocabulary learning.

Several studies have confirmed that the adoption of multimedia glosses promote leaners' vocabulary knowledge (e.g., Abuseileek, 2011; Rouhi and Mohebbi, 2012; Ramezanali and Faez, 2019; Ouyang et al., 2020). However, the results are inconsistent regarding the effectiveness of different glossing modes on vocabulary learning. AI-Seghayer (2001) and Lin and Tseng (2012) reported that the glossing of text and video was more effective in learning unfamiliar words than that of text and picture, whereas Chun and Plass (1996) found the opposite results and Akbulut (2007) concluded that there was no significant difference between these two glosses. In addition, extensive research has already reported the effective role of adding a still picture to clarify the word's meaning or a video in which the learner performs a gesture to the word in vocabulary learning (e.g., Tellier, 2008; Morett, 2019; Andrä et al., 2020). Nevertheless, the results were also mixed concerning which way was better. Mayer et al. (2015) and Repetto et al. (2017) found that adding a video in which the learner performed a gesture to the word led to better learning of words than adding a picture. However, Morett (2019) findings showed that students learnt concrete words better when viewing still images than those viewing iconic gestures (conveyed via video). On the other hand, Andrä et al. (2020) concluded that learning foreign language vocabulary with gestures (conveyed via dynamic video) was as effective as with pictures (conveyed via still images) in primary school contexts. Moreover, there are also inconsistencies reported on the efficacy of audio glossing presentation. Some studies revealed that students who were exposed to audio glosses achieved higher vocabulary scores than those having access to textual glosses (Rassaei, 2018; Ramezanali and Faez, 2019). However, the findings of research conducted by Kaplan-Rakowski and Loranc-Paszylk (2019) and Yeh and Wang (2003) concluded that the provision of audio glosses, such as word pronunciation, was not conducive to vocabulary learning and retention. In addition, there have been inconclusive results regarding reading comprehension (Ariew and Ercetin, 2004; Bowles, 2004; Lee and Lee, 2015; Taylor, 2020). Brandl (2002, p. 87) points out “whereas many educators enthusiastically embrace the use of internet-based reading materials, little theoretical and empirical research exits that demonstrates how to make use of such practices in a sound pedagogical way.” Thus, the purpose of this research is to explore the effects of multimedia glossing modes, particularly text, audio, videos, and pictures, on EFL learners' vocabulary learning and reading comprehension.

## Literature Review

### Multimedia Glossing

A gloss stands for brief definitions or translations of the new words, either in L1 or L2, which is available in the text (Nation, 2013). Lomicka (1998, p. 41) thinks that “Glosses are most often supplied for “unfamiliar” words, which may help to limit continual dictionary consultation that may hinder and interrupt the L2 reading comprehension process.”

With the development of computer technology, multimedia glosses came into use. Compared with traditional glosses, multimedia glosses have several advantages. Firstly, traditional glosses mainly include textual information, whereas computer-assisted glosses can take various forms, e.g., videos, pictures, and audio (Abuseileek, 2011). Besides, the combination of various multimedia glossing modes can be presented in computer-assisted glosses. For instance, in AI-Seghayer (2001)'s research, learners from three groups encountered a different version of glossing: “a version with L2 definition only, a version with L2 definition coupled with still pictures, and a version with L2 definition plus dynamic videos.” Secondly, different from traditional glosses, which usually exist in the margin of the text, hypermedia glosses can be linked to the glossed word and can appear in different locations, such as in a pop-up window or at the end of the text.

### Cognitive-Affective Theory of Learning With Media

Multimedia learning environments were defined as learning environments where learners adopt two different modes to acquire the knowledge, namely verbal and nonverbal (Moreno and Mayer, 2007). The cognitive-affective theory of learning with media (CATLM) proposed by (Moreno, 2005), was the extension of Mayer's cognitive theory of multimedia learning. It emphasizes that besides the design of multimedia instructions, some external factors, such as learners' motivation, strategies, or affect, play an essential role in multimedia learning.

CATLM is based on the seven assumptions, but four assumptions that are related to our study were illustrated:

1. The dual-channel assumption, based on Dual Coding Theory (Paivio, 1986), suggests that people have separate channels for processing various information modalities;
2. The limited capacity assumption, based on Cognitive Load Theory (Sweller, 1999), suggests that people can only process a few pieces of information at any one time within each channel;
3. The active processing assumption proposes that meaningful learning occurs when the learner puts the effort into cognitive processes, including selecting, organizing, and integrating new information with the prior knowledge (Mayer and Moreno, 2003); and
4. Motivational factors mediate learning through enhancing or lessening cognitive engagement (Pintrich, 2003).

According to Moreno and Mayer (2007), CATLM suggests that instructional media is recommended to contain both verbal explanations, including spoken words, and nonverbal knowledge presentations, such as pictures and sounds. In addition, meaningful learning occurs when people can attend to and choose related verbal and nonverbal information, then organize them into a coherent mental model and finally integrate the information with their existing knowledge. Furthermore, the presentation of words and pictures simultaneously allows people to mentally relate the two representations because they are stored in separate working memory, reducing cognitive load (Mayer, 2009).

### Efficacy of Multimedia Glossing on Vocabulary Learning and Reading

#### Efficacy of Multimedia Glossing on Vocabulary Learning

Extensive research has been carried out on the effects of multimedia glossing on learners' vocabulary acquisition, and most of the studies suggest that the addition of multimedia glossing facilitates learners' vocabulary learning. In a recent study, Ouyang et al. (2020) conducted an eye-tracking study to investigate the effectiveness of multimedia glosses on incidental word learning. Forty-five high-intermediate EFL learners were assigned to two conditions: textual gloss and no glosses. Their eye movements were recorded. Unannounced vocabulary tests were given to measure their recall and recognition of the words. The results showed that compared with students without glosses, learners with textual glosses performed better in both vocabulary tests. What is more, the attention given to glossing and words promoted the intake of the new words. Likewise, the research conducted by AlRamadhan (2020) and Rouhi and Mohebbi (2012) also showed that students who received textual glosses outperformed their counterparts without glosses.

Although numerous studies have revealed that glossing presentation benefited vocabulary learning, the results are inconclusive regarding the effectiveness of different glossing modes and whether adding an additional glossing mode to single gloss can enhance vocabulary learning (Ramezanali and Faez, 2019; Ramezanali et al., 2020).

When it comes to whether picture glosses are more effective than video glosses, the results are inconclusive. Akbulut (2007) recruited 69 advanced EFL students to investigate the effects of three hypermedia glosses on learners' vocabulary learning, namely, definitions of words only, definitions with videos, and definitions with pictures. The participants were given a vocabulary pretest, posttest, and delayed test. The results indicated that both groups who were presented with definitions coupled with pictures and videos achieved significantly higher scores on vocabulary tests than the definition alone group, but there is no significant difference among the picture and video group. Likewise, Rouhi and Mohebbi (2013) compared the effectiveness of video and pictorial glosses, and the results also revealed there was no significant difference between picture and video glossing on vocabulary learning. On the contrary, AI-Seghayer (2001) adopted a within-subject design and aimed at investigating which of the image modalities, namely still pictures and vivid videos, are more beneficial for ESL learners' vocabulary learning. The author came to the conclusion that the presentation with videos is more effective than that of pictures. Chun and Plass (1996) also investigated the effect of visual glosses on German students' incidental vocabulary learning. The results revealed that students who had access to text + pictures achieved significantly higher scores than those who were presented with text + videos and text only.

Regarding whether adding pictures to marginal glossing would aid L2 learners' vocabulary retention, the results are also controversial. Ramezanali et al. (2020) performed a meta-analysis of 22 studies, which included only between-subjects design, to compare the effectiveness of multimedia single vs. dual glossing on students' vocabulary acquisition. The results indicate that dual glossing modes (e.g., text plus picture) had a moderate effect size than single glosses (e.g., L1 or L2 glosses alone) and suggest that adding an additional glossing (e.g., pictorial glossing mode) to single textual glosses promotes learners' word learning. However, unlike previous studies in which the superiority of text-plus-picture glossing modes over text only glosses were found (e.g., Kost et al., 1999; AI-Seghayer, 2001; Yoshii and Flaitz, 2002), other studies found the opposite results (e.g., Boers et al., 2017b; Rungwaraphong, 2020). Rungwaraphong (2020) indicated that although Thai EFL students were more likely to choose picture-only and picture-and-text glosses than textual-only glosses, students who used textual-only glosses were more successful at interpreting the unfamiliar words and achieved higher scores in vocabulary test. The reason was that depending on pictures only involved some risks which may cause misinterpretation.

With respect to audio glosses, there are still inconsistencies reported on some studies. Using a between-participant design, Kim and Gilman (2008) investigated the efficacy of adding spoken texts on Korean students' vocabulary learning. The results indicate that adding spoken text did not lead to students' vocabulary gains because it caused an unnecessary distraction. Kaplan-Rakowski and Loranc-Paszylk (2019)'s study also obtained the same results. In contrast, some studies showed that audio glosses seem to be more useful than textual glosses for promoting vocabulary learning (e.g., Rassaei, 2018; Ramezanali and Faez, 2019).

#### Previous Research of Multimedia Glosses and Reading Comprehension

Considerable research has been directed at the role of text, picture, video, and audio glosses in reading comprehension, but there is controversy respecting the effectiveness of multimedia glossing on learners' reading comprehension. On the one hand, both meta-analytic studies conducted by Abraham (2008) and Taylor (2020) revealed that learners with CALL glosses tended to comprehend the text more effectively than those without access to glosses. And Taylor (2020) findings further found that textual plus visual glossing mode, including pictures or videos, was the most effective way to improve learners' reading comprehension.

On the other hand, other studies revealed that the use of multimedia glosses did not facilitate learners' reading comprehension. Ariew and Ercetin (2004)'s study aims to explore whether different multimedia glosses facilitate reading comprehension. A total of 84 adult ESL students took part in the study and they were asked to use multimedia glosses while reading the hypermedia text. The results showed that the use of multimedia glosses was not beneficial for participants' reading comprehension and video annotations had a negative effect on their reading comprehension. The study conducted by Sakar and Ercetin (2005) explores the effectiveness of multimedia annotation on reading comprehension. Participants were 44 EFL adults learners. Through quantitative and qualitative analysis, the results indicate that although participants showed a stronger preference for video annotation to textual and audio glosses, the annotation use, including audio, video, and textual glosses, negatively affects learners' reading comprehension.

Thus, due to the inconsistency of results concerning the effectiveness of different multimedia glossing presentation on both students' vocabulary acquisition and reading comprehension, it is of great significance to explore whether hypermedia gloss presentation in different modes (text, pictures, audio, and videos) facilitates EFL learners' vocabulary acquisition and reading comprehension. In addition, based on Yanagisawa et al. (2020)'s meta-regression analysis, there are few studies including auditory glosses, so it is quite necessary to evaluate the effects of audio glossing modes. It is also noteworthy that few studies have tackled the above issues in the Chinese EFL context. Most of the early studies were mostly short-term, such as 1 or 2 weeks only; the present study, which is longer-term, lasted for one semester. To bridge the identified gap, the present study is aimed at investigating the effectiveness of different multimedia glossing modes, including L2 definition alone, L2 definition coupled with audio, L2 definition plus picture, and L2 definition with video glossing mode, on EFL learners' vocabulary acquisition and reading comprehension in the Chinese EFL context.

The research questions that will be addressed in the present study are as follows:

1. RQ 1. Which multimedia glossing type (L2 definition only, L2 definition coupled with audio, L2 definition plus video clips, and L2 definition coupled with picture) has a significant effect on EFL students' vocabulary learning?
2. RQ 2. Which multimedia glossing type (L2 definition only, L2 definition coupled with audio, L2 definition plus video clips, and L2 definition coupled with picture) has a significant effect on EFL students' reading comprehension?
3. RQ 3. What are students' perspectives on these four glossing modes?

## Methods

This research adopted a between-participant design. It is also a mixed methods research containing both qualitative and quantitative data.

### Participants

The participants consisted of 160 EFL learners who were all English major students at a university in China. There were 15 men and 145 women. The median age was 18.9 years. The average time that the participants had learnt English was 12.3 years. In a between-subject design, 37 students served in L2 definition only group, 42 students in L2 definition and video group, 41 in L2 definition and audio group, and 40 participants in L2 definition and picture group.

### Computerized Texts

The experiment texts were eight passages with about 350 words each, chosen from one of standardized English tests in China-CET 4.

#### Pilot Test of the Experiment Text

Before the actual implementation of the research, 20 students with similar language proficiency with the participants were selected to read the texts and were encouraged to underline all the unknown words. If more than half of the students marked the word as unknown, it was selected to be glossed.

#### Glossing Format

Approximately 10 words in each passage were glossed. All the glossed words were marked in blue and hyperlinked, and when the participants clicked the words, a window popped up. In L2 definition group, participants not only can see the definition of the words, but also synonyms, antonyms, or example sentences. Besides the same contents as the L2 definition-only group, in L2 definition coupled with audio group, students can also listen to the pronunciation and the definition of the words. In L2 definition with video glossing mode group, students can also watch video clips. In L2 definition plus picture groups, learners can also view a picture to elucidate the word's meaning.

### Assessment Tasks

#### Vocabulary Pre-/Post-tasks

A vocabulary test was designed to examine the effectiveness of four glossing modes, and this test was utilized as both pre- and post- vocabulary tests tests. In order to provide a more natural learning environment, participants were not informed in advance that they would take the test.

The vocabulary test included recognition tasks and production tasks. The production tasks had 15 items, and the recognition tasks were 45-item multiple-choice questions. In addition, the vocabulary production tasks were given prior to the recognition tasks. For production tasks, students were required to write L1 equivalents or synonyms of the given words. For the recognition tasks, students were required to select the right words from the other three distractors based on the definition provided by the stem. What is more, so as to eliminate the effect of guessing, an option “I do not know” was included to each item.

#### Pre-/Post-reading Comprehension Tasks

The pre- and post- reading comprehension tasks tasks were the same and made up of five multiple-choice comprehension questions for each passage, with 40 questions in total. The questions were directly chosen from the CET 4. In order to eliminate the influences of the pretest on posttest, the time span during these two tests was more than 1 month. Moreover, so as to have a better understanding of the efficacy of glosses on reading comprehension, the reading comprehension questions were divided into two parts and were analyzed separately. Part 1 included 16 questions which need to be answered by using the knowledge of the glossed words directly. Part 2 consisted of 24 questions which need not be answered using the glossed words directly.

Both the vocabulary and reading comprehension tests were scored in a binary fashion. Students got 1 point for a correct answer and 0 point for a wrong answer. The highest possible score on vocabulary test was 60 and on reading comprehension test was 40. All tests were completed online instead of using a traditional paper-and-pen format.

#### Questionnaire and Interview

All the participants were invited to fill in a questionnaire after the whole experiment. The aim of the questionnaire was to get information about participants and their experience with using multimedia glossing while reading hypermedia texts. Besides the demographic information, the questionnaire asked students (1) which glossing modes helped them learn the words and read the texts easily and (2) which glosses increased their motivation to learn more words and read the texts and (3) how helpful the multimedia glosses were in learning new words and enhancing their reading comprehension.

Then 20 volunteers took part in interviews a day after the experiment. The semistructured interview was on a one-on-one basis and lasted for about 15 minutes for each participant. The purpose of the interviews was to obtain comprehensive data regarding participants' use of multimedia glosses while reading the computerized text.

### Procedure

The whole experiment lasted for one semester, namely 3 months. One month prior to the experiment, the students were given the prevocabulary test and prereading comprehension tests. During the phase of experiment, in week 1, participants from four classes were assigned randomly to the four treatment groups. First, the teachers explained the aim and the process of the study and asked students to sign the informed consent form. Then followed by the next phase, which took place in the lab, students read the computerized text using different multimedia glossing modes and took the reading comprehension tests after learning each reading passage. After students learnt all the eight passages, they were required to take the postvocabulary test. After these procedures, students were free to choose the multimedia glossing modes as they want, so they can experience different modes which were different from that they used during the intervention. Then they were required to complete the questionnaire and finally, 20 participants attended the interview.

## Results

### Effects of Different Glossing Modes on Vocabulary Learning

#### Effects of Different Glossing Modes on Vocabulary Production Tasks

As shown in Table 1, all the four glossing groups earned relatively similar scores on the vocabulary production pretest (L2 definition group, *M* = 3.08; L2 definition plus video group, *M* = 3.95; L2 definition plus picture group, *M* = 3.30; L2 definition plus audio group, *M* = 3.85). However, there were larger differences among the groups on the vocabulary production posttest. To be more specific, the means of the L2 definition plus picture group were highest, followed by L2 definition plus video group and L2 definition + audio group; the L2 definition group was lowest, and all the three combination of L2 definition and other modes were higher than the mean of the L2 definition only group. In order to determine whether these differences were statistically significant, a 4 (glossing mode) × 2 (time: pretest and posttest) mixed-design ANOVA was conducted. The results indicated that the differences were statistically significant for both time, *F*_(1,156)_ = 748.701, *p* < 0.05, η^2^ = 0.828 and group (glossing mode), *F*_(3,156)_ = 13.341, *p* < 0.05, η^2^ = 0.204. In addition, the interaction between glossing modes and time of measurement was significant, *F*_(3,156)_ = 32.431, *p* < 0.05, η^2^ = 0.384, suggesting that means of the groups differed depending on the time of measurement. Because of a significant interaction effect between glossing modes and time of measurement, simple main effects should be carried out.

**Table 1:** Means and standard deviations for vocabulary production pre- and post-tests.

**Treatment**	**Vocabulary production**					
	**Pretest**			**Posttest**		
	***N***	**Mean**	**Std. deviation**	***N***	**Mean**	**Std. deviation**
L2 definition	37	3.08	2.11	37	6.19	3.19
L2 definition + video	42	3.95	3.05	42	11.24	2.90
L2 definition + picture	40	3.30	2.38	40	12.63	3.12
L2 definition + audio	41	3.85	2.60	41	9.24	3.63
Overall	160	3.56	2.57	160	9.91	3.98

The simple main effects of time measures indicated that students from all of the four groups earned significantly better scores on the posttest than the pretest (see Table 2). The simple main effects of glossing types (see Table 3) presented that four glossing groups did not have significant differences in pretest. However, the results of the posttest had significant differences (see Table 3). The L2 definition and picture groups achieved the highest scores, followed by L2 definition plus video, the L2 definition and audio group ranked next and the L2 definition only group was the lowest. But there was no significant difference between L2 definition plus picture and L2 definition coupled with video group.

**Table 2:** The simple main effect of time measurement on vocabulary production tasks.

**Group**	**(I) Time**	**(J) Time**	**Mean difference (I–J)**	**Std. error**	**Sig**.
L2 definition only	1	2	−3.108	0.477	<0.001
L2 definition plus video	1	2	−7.286	0.477	<0.001
L2 definition plus audio	1	2	−5.390	0.453	<0.001
L2 definition plus picture	1	2	−9.325	0.458	<0.001

“*1” refers to pretest; “2” refers to posttest*.

**Table 3:** The simple main effect of glossing mode on production pre- and post-tests.

**Time**	**(I) Group**	**(J) Group**	**Mean difference (I–J)**	**Std. error**	**Sig**.
Pretest	L2 definition only	+ video	−0.871	0.580	0.581
	L2 definition only	+audio	−0.773	0.583	0.712
	L2 definition only	+ picture	−0.219	0.587	0.999
	+video	+ audio	0.099	0.565	>0.999
	+video	+ picture	0.652	0.568	0.826
	+ audio	+ picture	0.554	0.572	0.913
Posttest	L2 definition only	+ video	−0.5.049	0.726	<0.001
	L2 definition only	+audio	−3.055	0.730	<0.001
	L2 definition only	+ picture	−6.436	0.735	<0.001
	+video	+ audio	0.1.994	0.707	0.032
	+video	+ picture	−1.387	0.712	0.279
	+ audio	+ picture	−3.381	0.716	<0.001

**Figure 1:**
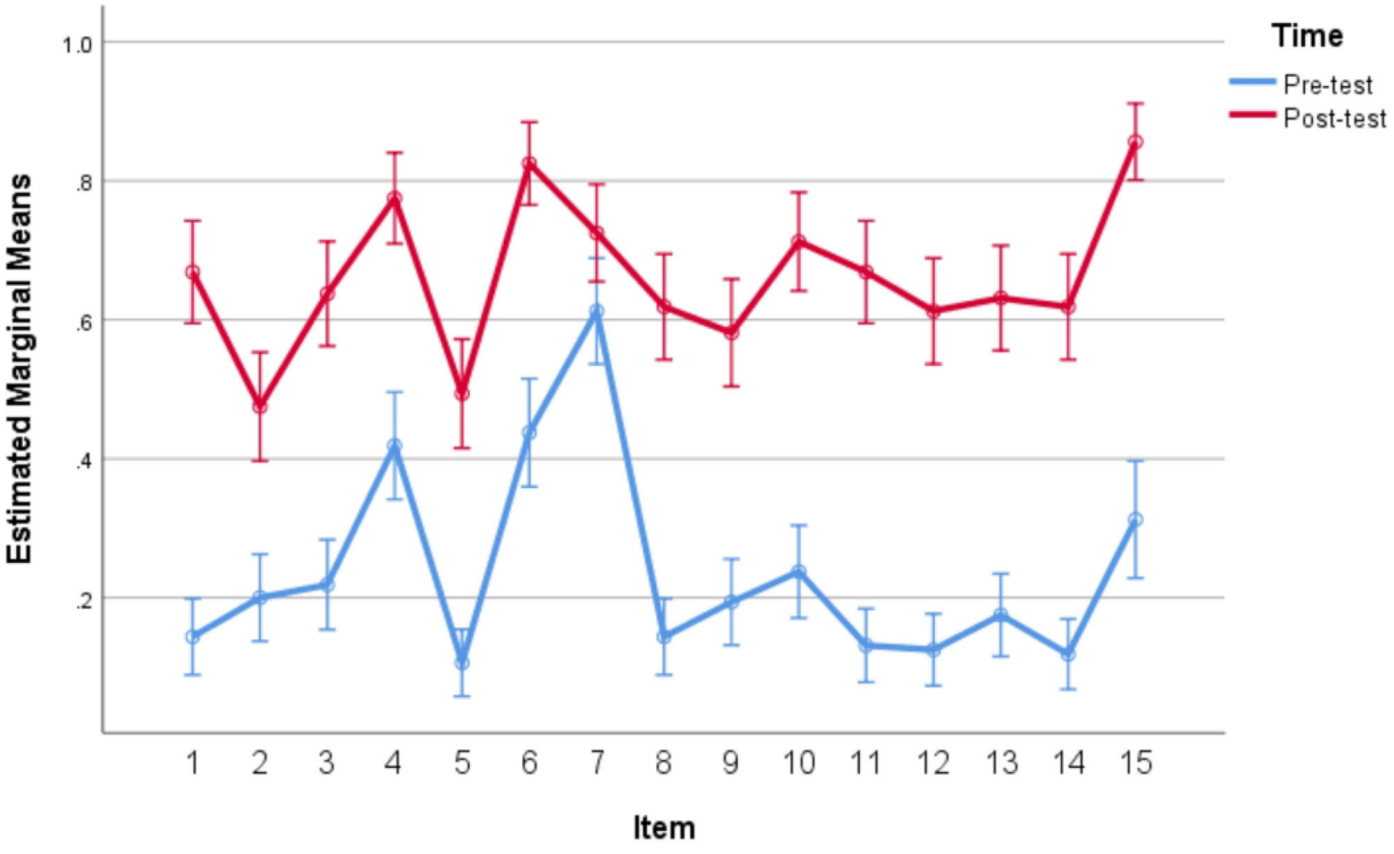
Item analyses for vocabulary production test.

Besides the participant analyses conducted above, item analyses were also conducted by using a within-subject repeated measures ANOVA in which both item and time were entered as within-subject factors. The item analysis yielded significant main effects for both items, *F*_(14,146)_ = 28.347, *p* < 0.05, η^2^ = 0.731, time, *F*_(1,159)_ = 417.604, *p* < 0.05, η^2^ = 0.724 and the interaction of item and time, *F*_(14,146)_ = 5.847, *p* = 0.040 < 0.05, η^2^ = 0.359. The results of item analysis of vocabulary production tasks can be visually showed on Figure 1.

#### Effects of Different Glossing Modes on Vocabulary Recognition Tasks

Concerning the vocabulary recognition pretests, as shown in Table 4, all the four glossing groups also achieved relatively similar scores (L2 definition group, *M* = 14.00; L2 definition plus video group, *M* = 15.86; L2 definition plus picture group, *M* = 14.08; L2 definition plus audio group, *M* = 15.05). Whereas there were larger differences among the groups on the posttest. To be more specific, the means of the L2 definition plus picture group were highest (*M* = 32.45), followed by L2 definition plus video group and L2 definition + audio group, with 32.26 and 28.00, respectively; the L2 definition group was lowest (*M* = 21.76), and all the three combination of L2 definition and other modes were higher than the mean of the L2 definition only group. In order to check if statistically significant differences existed, a 4 (glossing mode) × 2 (time: pretest and posttest) mixed-design ANOVA was conducted. The results showed that the statistically significant differences were found for both time, *F*_(1,156)_ = 836.305, *p* < 0.05, η^2^ = 0.843 and group (glossing mode), *F*_(3,156)_ = 8.545, *p* < 0.05, η^2^ = 0.141. In addition, there was a significant interaction effect between glossing modes and time of measurement, *F*_(3,156)_ = 22.648, *p* < 0.05, η^2^ = 0.303, suggesting that means of the groups differed depending on the time of measurement. Because of a significant interaction effect between glossing modes and time of measurement, simple main effects should be carried out.

**Table 4:** Means and standard deviations for vocabulary recognition pre- and posttests.

**Treatment**	**Vocabulary recognition**					
	**Pretests**			**Posttests**		
	***N***	**Mean**	**Std. deviation**	***N***	**Mean**	**Std. deviation**
L2 definition	37	14.00	4.15	37	21.76	6.19
L2 definition+ video	42	15.86	6.57	42	32.26	7.33
L2 definition + picture	40	14.08	5.82	40	32.45	7.57
L2 definition + audio	41	15.05	6.60	41	28.00	7.43
Overall	160	14.78	5.91	160	28.79	8.29

The simple main effects of time measures indicated that students from all of the four groups earned significantly better scores on the posttest than the pretest (see Table 5). The simple main effects of glossing types (see Table 6) presented that four glossing groups did not have significant differences in pretest. However, the results of the posttest had significant differences (see Table 6). The L2 definition and picture groups achieved the highest scores, followed by L2 definition plus video, the L2 definition and audio group ranked next, and the L2 definition only group was the lowest. However, there was no significant difference between L2 definition plus picture and L2 definition coupled with video group.

**Table 5:** The simple main effect of time measurement on vocabulary recognition tasks.

**Group**	**(I) Time**	**(J) Time**	**Mean difference (I–J)**	**Std. error**	**Sig**.
L2 definition Only	1	2	−7.757	0.996	<0.001
L2 definition plus video	1	2	−16.405	0.935	<0.001
L2 definition plus audio	1	2	−12.951	0.947	<0.001
L2 definition plus picture	1	2	−18.375	0.958	<0.001

“*1” refers to pretest; “2” refers to posttest*.

**Table 6:** The simple main effect of glossing mode on recognition pre- and posttests.

**Time**	**(I) Group**	**(J) Group**	**Mean difference (I–J)**	**Std. error**	**Sig**.
Pretest	L2 definition only	+ video	−1.857	1.333	0.662
	L2 definition only	+audio	−1.049	1.341	0.968
	L2 definition only	+ picture	−0.075	1.349	>0.999
	+ video	+ audio	0.808	1.298	0.990
	+ video	+ picture	1.782	1.306	0.683
	+ audio	+ picture	0.974	1.314	0.975
Posttest	L2 definition only	+ video	−10.505	1.617	<0.001
	L2 definition only	+audio	−6.243	1.626	0.001
	L2 definition only	+ picture	−10.693	1.636	<0.001
	+ video	+ audio	4.262	1.575	0.044
	+ video	+ picture	−0.188	1.585	>0.999
	+ audio	+ picture	−4.450	1.594	0.035

**Figure 2:**
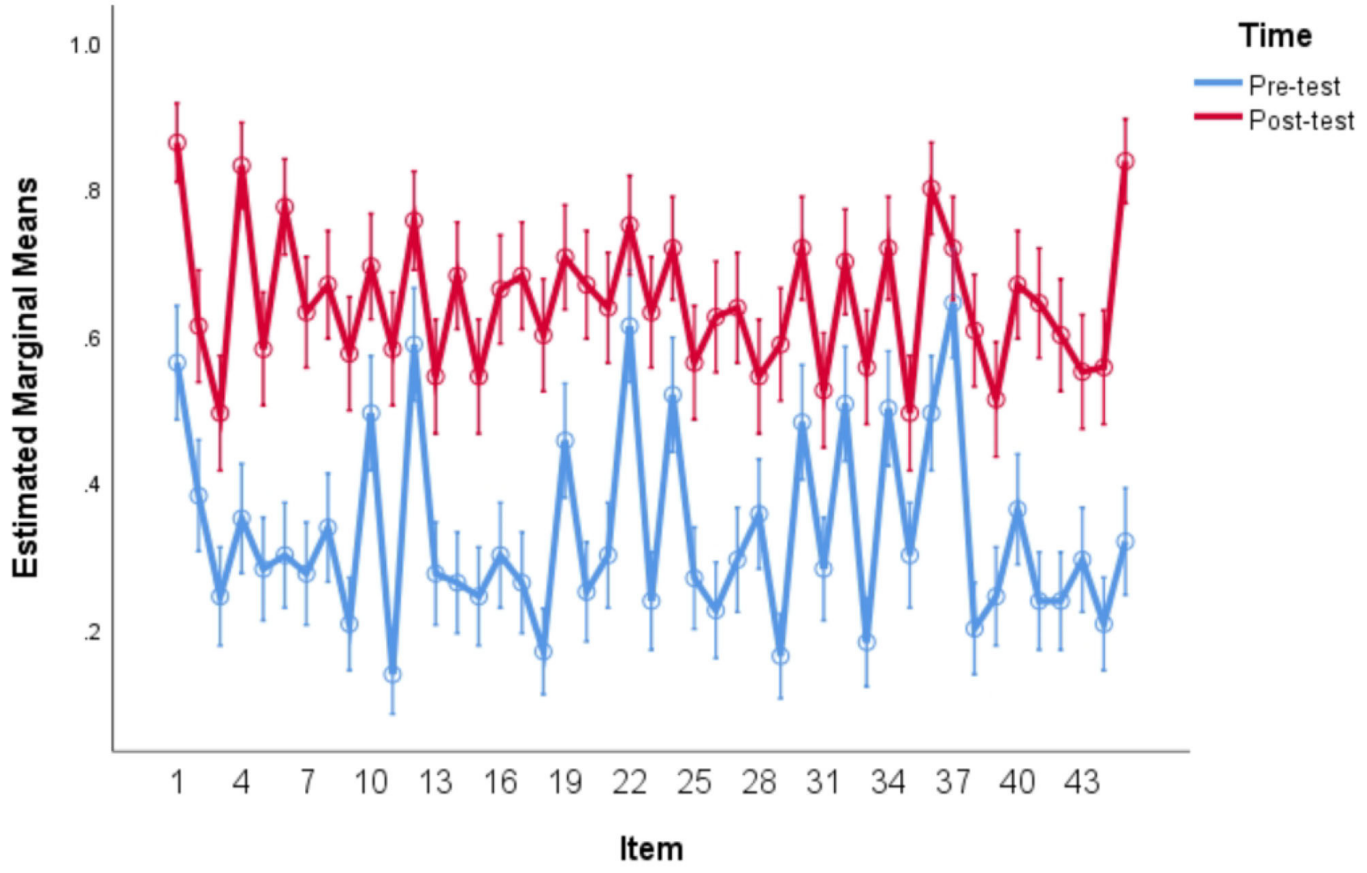
Item analyses for vocabulary recognition test.

Besides the participant analyses conducted above, item analyses were also conducted by using a within-subjects repeated measures ANOVA in which both item and time were entered as within-subjects factors. The item analysis yielded significant main effects for both items, *F*_(44,116)_ = 15.287, *p* < 0.05, η^2^ = 0.853, time, *F*_(1,159)_ = 384.558, *p* < 0.05, η^2^ = 0.707 and the interaction of item and time, *F*_(44,116)_ = 3.471, *p* < 0.05, η^2^ = 0.568. The results of item analysis of vocabulary recognition tasks can be visually showed on Figure 2.

### Results of Reading Comprehension Test

#### Effects of Different Glossing Modes on Reading Comprehension Tasks Which Required Glossed Words

As we mentioned above, all the 40 reading comprehension questions were divided into two parts and analyzed separately. First, the results of the reading comprehension questions that required the knowledge of the glossed words were shown below.

As Table 7 shows, all the four groups got relatively low reading comprehension scores on pretest and also for posttest. Regarding the posttest, the group had access to L2 definition plus video glosses had a slightly higher score than the other three groups. In order to check whether there were significant differences, a 4 (glossing mode) × 2 (time: pretest and posttest) mixed-design ANOVA was conducted. Results yielded only a significant main effect for time, *F*_(1,156)_ = 127.888, *p* < 0.05, η^2^ = 0.450. Neither significant main effect for glossing mode [*F*_(3,156)_ = 0.517, *p* = 0.763 > 0.05, η^2^ = 0.014] nor a significant interaction between time and glossing mode [*F*_(3,156)_ = 1.100, *p* = 0.351 > 0.05, η^2^ = 0.021] was found. The result concluded that for the reading comprehension questions that required the knowledge of the glossed words, all the four glossing modes promote students' reading comprehension, but there were no significant differences among these four groups.

**Table 7:** Means and standard deviations for reading comprehension which required glossed words pre- and posttests.

**Treatment**	**Reading comprehension which required glossed words**					
	**Pretest**			**Posttest**		
	***N***	**Mean**	**Std. deviation**	***N***	**Mean**	**Std. deviation**
L2 definition	37	7.19	3.35	37	10.51	3.92
L2 definition+ video	42	6.12	5.06	42	11.45	4.80
L2 definition + picture	40	6.05	4.34	40	10.72	4.55
L2 definition + audio	41	5.59	3.52	41	10.05	4.06

**Figure 3:**
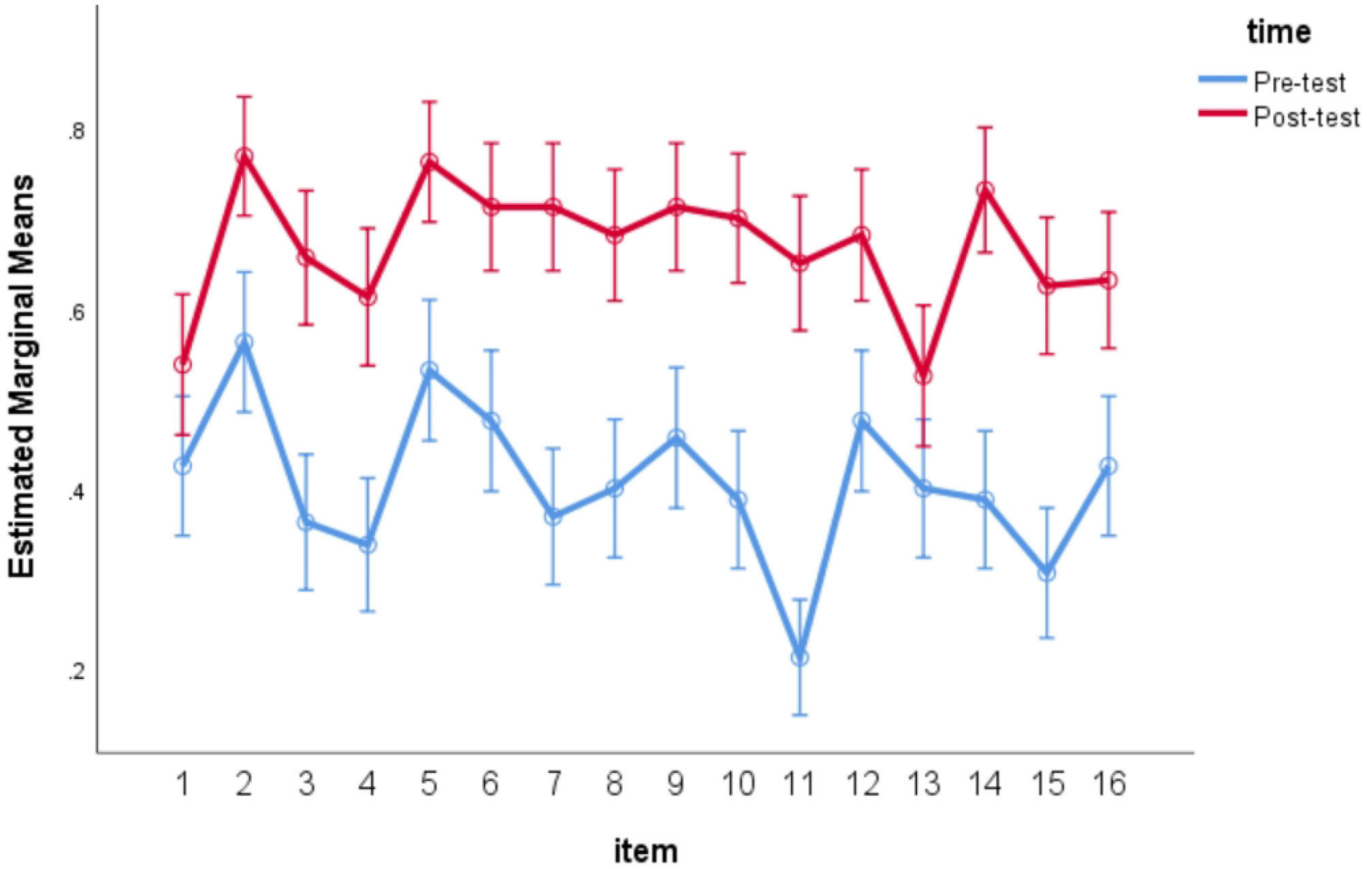
Item analyses for reading comprehension which required glossed words.

Besides the participant analyses conducted above, item analyses were also conducted by using a within-subject repeated measures ANOVA in which both item and time were entered as within-subject factors. The item analysis yielded significant main effects for both items, *F*_(15,145)_ = 9.000, *p* < 0.05, η^2^ = 0.482, time, *F*_(1,159)_ = 131.966, *p* < 0.05, η^2^ = 0.454 and the interaction of item and time, *F*_(15,145)_ = 2.657, *p* = 0.001 < 0.05, η^2^ = 0.216, as can be visually showed on Figure 3.

#### Effects of Different Glossing Modes on Reading Comprehension Tasks Which Did Not Require Glossed Words

Then, for the questions that did not require the knowledge of the glossed words, the results are shown below.

As Table 8 shows, all the four groups got relatively similar reading comprehension scores on pretest and also for posttest. Concerning the posttest, the group had access to L2 definition plus video glosses had a slightly higher score than the other three groups. In order to check whether there were significant differences, a 4 (glossing mode) × 2 (time: pretest and posttest) mixed-design ANOVA was conducted. Results yielded only a significant main effect for time, *F*_(1,156)_ = 42.135, *p* < 0.05, η^2^ = 0.213. Neither significant main effect for glossing mode [*F*_(3,156)_ = 0.880, *p* = 0.453 > 0.05, η^2^ = 0.017] nor a significant interaction between time and glossing mode [*F*_(3,156)_ = 1.067, *p* = 0.365 > 0.05, η^2^ = 0.020] was found. The result concluded that for the reading comprehension questions that did not require the knowledge of the glossed words, all the four glossing modes promote students' reading comprehension, but there were no significant differences among these four groups.

**Table 8:** Means and standard deviations for reading comprehension which did not require glossed words pre- and posttests.

**Treatment**	**Reading comprehension which did not require glossed words**					
	**Pretest**			**Posttest**		
	***N***	**Mean**	**Std. deviation**	***N***	**Mean**	**Std. deviation**
L2 definition	37	10.32	2.68	37	12.38	3.29
L2 definition+ video	42	11.04	3.39	42	13.12	3.39
L2 definition + picture	40	10.50	2.82	40	12.00	3.12
L2 definition + audio	41	10.90	2.69	41	11.88	2.98

**Figure 4:**
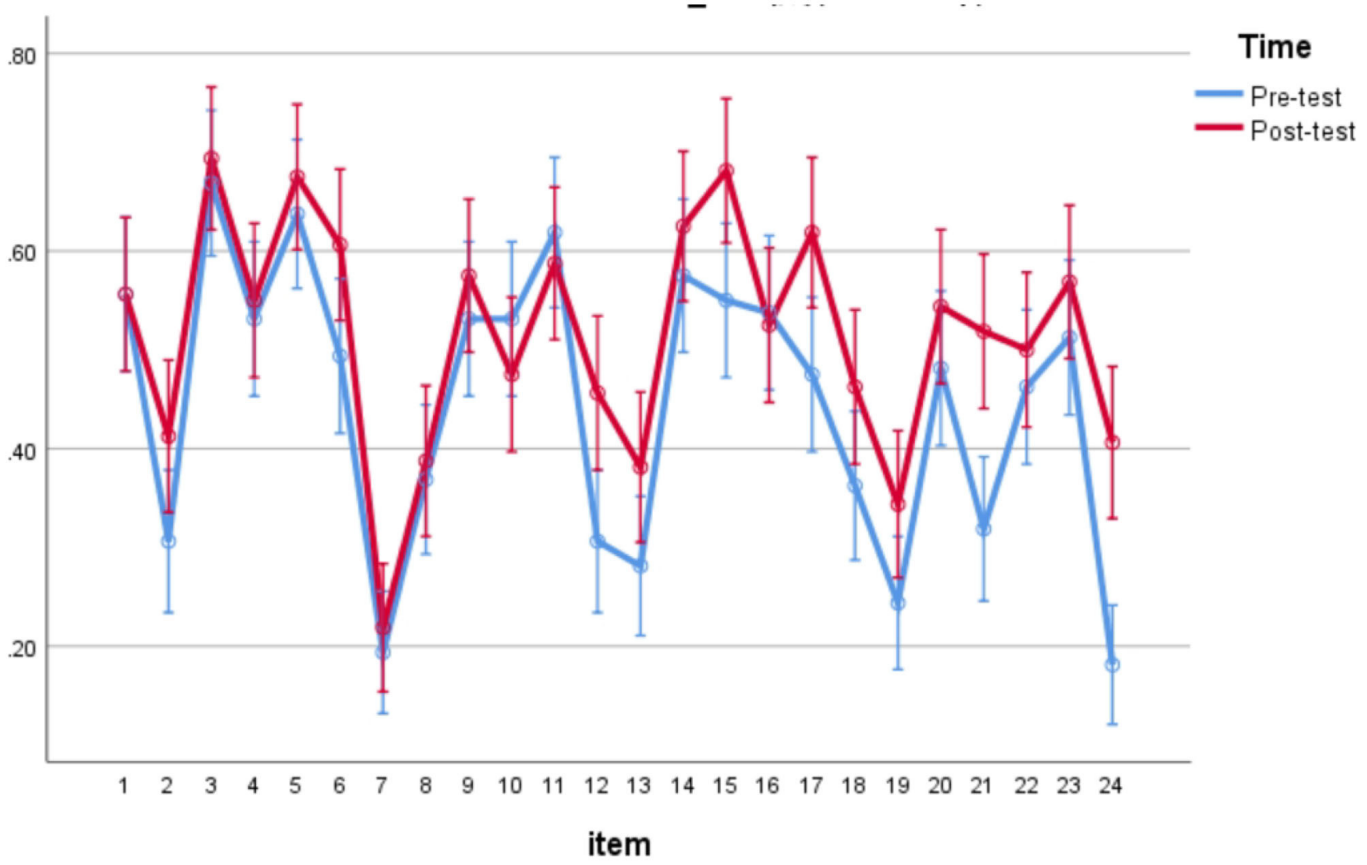
Item analyses for reading comprehension which did not require glossed words.

Besides the participant analyses conducted above, item analyses were also conducted by using a within-subject repeated measures ANOVA in which both item and time were entered as within-subject factors. The item analysis yielded significant main effects for both items, *F*_(23,137)_ = 24.330, *p* < 0.05, η^2^ = 0.803, time, *F*_(1,159)_ = 39.398, *p* < 0.05, η^2^ = 0.199 and the interaction of item and time, *F*_(23,137)_ = 2.496, *p* = 0.001 < 0.05, η^2^ = 0.295, as can be visually shown on Figure 4.

### Results of Questionnaire and Interviews

All the 160 participates completed the questionnaire. Firstly, the questionnaire asked students which glossing modes helped them learn words easily and which glosses increased their motivation to learn more words and read the texts. Each question was duplicated four times for each glossing mode and students were required to rank their response on a 5-point Likert scale (1 = strongly agree; 5 = strongly disagree). As shown in Table 9 (questions 1–4), most students took the view that the dual glossing modes of L2 definition and video and picture help them learn the words easily (59.4% and 56.25%, respectively), followed by L2 definition plus audio glosses (40%), and lastly the L2 definition-only glosses (18.75%).

**Table 9:** Participants' attitudes towards the glossing modes.

**Questions**	**L2 definition only**		**L2 definition +picture**		**L2 definition +video**		**L2 definition +audio**	
	**Percentage**	***n***	**Percentage**	***n***	**Percentage**	***n***	**Percentage**	***n***
1–4 It is easy for me to learn new words with this mode.	18.75%	30	56.25%	90	59.4%	95	40%	64
5–8 I feel motivated when I use this mode to learn words and read the texts.	18.75%	30	68.75%	110	75.63%	121	41.25%	66

*Each question was duplicated four times for every glossing mode and n stands for the number of subjects' responses who either strongly agreed or agreed to the sentence*.

When asked which glossing mode increased their motivation to learn more words and read the texts (questions 5–8), 75.63% and 68.75% participants strongly agreed or agreed that the addition of L2 definition plus video and picture glossing mode increased their motivation. For L2 definition plus audio glosses, 41.25% students strongly agreed and agreed that they felt motivated when using this mode. Lastly, 18.75% students strongly agreed and agreed that L2 definition only glossing mode motivated them to learn words and read the texts.

Participants were then asked to rate the helpfulness of each of the glossing modes in enhancing vocabulary learning and reading comprehension on a scale of 1–5 (1 = very helpful, 5 = not at all helpful). The results are presented in Table 10.

**Table 10:** Results on the usefulness of the glosses.

	**Frequency** **(L2 definition only)**	**Frequency** **(+ picture)**	**Frequency** **(+ video)**	**Frequency** **(+ audio)**
Extremely helpful	36 (22.5%)	50 (31.25%)	63 (39.375%)	50 (31.25%)
Helpful	54 (33.75%)	92 (57.5%)	77 (48.125%)	68 (42.5%)
Somewhat helpful	32 (20%)	5 (3.125%)	5 (3.125%)	19 (11.875%)
Neutral	37 (23.125%)	13 (8.125%)	15 (9.375%)	22 (13.75%)
Unhelpful	1 (0.625%)	0	0	1 (0.625%)
Mean	2.46	1.88	1.83	2.1

The findings in Table 10 revealed that altogether, nearly 88.75% and 87.5% of the learners considered that the addition of L2 definition coupled with picture and video glossing extremely helpful or helpful. It is worth noting that nobody thought using L2 definition + picture and video unhelpful (0%); what is more, 73.75% participants considered the L2 definition coupled with audio glossing extremely helpful or helpful, and also nearly half of the participants regard L2 definition-only glossing presentation helpful.

In sum, the questionnaire data reflected that students showed positive attitudes towards using the glossing modes, and they considered L2 definition plus visual modes, including video and picture, most useful, followed by L2 definition plus audio and finally L2 definition only. Most students thought that the addition of L2 definition coupled with video and picture glosses helped them learn the words and read the texts easily and most of them also agreed that the addition of visual glossing modes motivated them to learn the words and read the texts.

The last data was from interview and altogether 20 students joined the one-on-one interviews in order to gain a deeper understanding of students' perspectives on using the multimedia glosses. Almost all the interviewees thought that the L2 definition and video and picture glossing modes were the most effective and most favorable. The reason is that combining pictures and videos can motivate students to learn words vividly and can clarify the meaning of the new words. The following excerpts were from two of the interviewees:

Since I was in middle school, I just got used to retaining the vocabulary by using the wordlists, which was so boring. I like the new ways of memorizing words and they can help me know the meaning of the words while reading. It is quite convenient. Through L2 definition and picture glossing mode, I can understand the meaning of the words clearly, and it left a deep impression on me (Excerpt 1).
Among these four glossing modes, I prefer L2 definition and video. The videos are quite dynamic and vivid, which greatly motivated me to learn and attracted my attention. I think in the future, I will continue trying these ways of retaining words (Excerpt 2).

## Discussion

In relation to research question 1, the results first showed that all the four groups got higher scores on the posttest than those on pretests on both vocabulary production and recognition tasks, suggesting the effectiveness of multimedia glosses on vocabulary acquisition. In previous research (e.g., Abuseileek, 2011; Rouhi and Mohebbi, 2012; Ramezanali and Faez, 2019; Ouyang et al., 2020), the positive effects of multimedia glosses on vocabulary tests have also been found. In addition, students in all four groups achieved better gains on vocabulary recognition tests (overall gains: 14.01, from 14.78 to 28.79) than on vocabulary production tests (overall gains: 6.35, from 3.56 to 9.91), showing that multimedia glosses facilitate learning knowledge of recognition more than learning of recall knowledge. These results were also found in some other studies, such as Ramezanali et al. (2020) and Yun (2011). Since learning of knowledge of recognition comes before the acquisition of recall knowledge (González-Fernández and Schmitt, 2020), the addition of glosses maybe facilitates learning of recognition faster and more readily than that of recall (Ramezanali et al., 2020).

Then our results also indicated that students who were exposed to dual glossing modes, including L2 definition + picture, L2 definition + video and L2 definition + audio, outperformed those participants who had access to single glossing mode, namely L2 definition only, on both vocabulary production and recognition tests. These results are in line with the previous research that has shown that dual glossing modes were superior to single glossing modes (AI-Seghayer, 2001; Akbulut, 2007; Abraham, 2008; Yun, 2011; Rassaei, 2018).

One plausible explanation is that according to cognitive-affective theory of learning with media, especially the modality effect, which refers to “a cognitive load learning effect that occurs when a mixed-mode (partly visual and partly auditory) presentation of information is more effective than a single-mode (either visual or auditory alone) presentation of the same information” (Low and Sweller, 2014, p. 227). It is because when learners were exposed to dual glossing modes, they are able to encode the glossed vocabulary in both visual and verbal formats and they can process it in both verbal and visual two channels (Ramezanali et al., 2020). It induces a low cognitive load by splitting it between the visual and verbal channels because auditory and visual materials are separately processed in their own system. Thus, “the total load is spread between the visual and the auditory components in the working memory system” (Low and Sweller, 2014, p. 235). So students can learn better in the learning environment where learners are presented with information both visually and verbally, than those who have access to only information in a single mode (Mayer, 2017).

Another possible interpretation for the results is that providing dual multimedia glossing catches more and longer attention to the glossing words than single glosses (Boers et al., 2017a). Since the amount of attention on unfamiliar words is a vital predictor of vocabulary learning (Schmitt, 2008), the groups presented with dual glosses are more likely to attend to the vocabulary, thereby creating stronger memory traces (Boers et al., 2017a).

Moreover, the present research also concluded that both two visual groups, namely L2 definition coupled with picture and L2 definition plus video, had an advantage over the L2 definition plus audio group in acquisition of vocabulary. These findings are consistent with previous research that has found the provision of textual plus video or picture glosses more beneficial for vocabulary learning than textual coupled with audio glosses (e.g., Kim and Gilman, 2008; Ramezanali and Faez, 2019).

Based on the questionnaire and interview data, it can be seen that students who were exposed to visual materials, irrespective of still pictures or dynamic videos, were prone to be more motivated and stimulated to learn unfamiliar words because providing visual materials has several advantages, such as making materials appealing, and elucidating complex concepts, or procedures and helping learners visualize the definition more meaningfully (Kim and Gilman, 2008; Mayer, 2009). The combination of visual information tended to make the meaning of unknown words clear, and it did not lead to any additional cognitive load (Mayer et al., 2014). The following statements show that students had positive attitudes toward L2 definition coupled with picture and video glosses. “I'm highly motivated to learn vocabulary by watching videos, and retaining words is not a burden for me now. It helps me have a deeper understanding of the words and left a clear impression on me.” “Watching pictures and videos helps me visualize the meaning of the vocabulary and they are quite vivid.” In contrast, audio seems to be more transient since students are exposed to the words only when they are presented (Leahy and Sweller, 2011; Singh et al., 2012). Furthermore, another possible interpretation is that students were freshmen and just graduated from high school, where the teachers paid more attention to improve students' reading and grammar skills, rather than listening skills. They also got used to retaining words without knowing how words are pronounced. Thus, the L2 definition coupled with audio glosses caused them an unnecessary distraction.

In response to research question 2, the findings from this study showed that no matter for the reading comprehension questions that required the knowledge of the glossed words or for the questions that did not require the knowledge of the glossed words directly, all the multimedia glossing presentation promotes EFL learners' reading comprehension. Moreover, in comparison with the questions that did not directly rely on the knowledge of the glossed words (gains: 1.64, from 10.71 to 12.35), students made bigger improvements for the reading comprehension questions that directly required the glossed words (overall gains: 4.48, from 6.21 to 10.69). The findings coincide with previous research, suggesting that the multimedia glosses are beneficial to text comprehension (Blohm, 1982; Davis, 1989; Lomicka, 1998; Taylor, 2020). The meta-analysis conducted by Taylor (2020) concluded that 80% of L2 readers who are exposed to multimedia glosses comprehend the text better than students without CALL glosses, with an overall effect size of 0.84. Rott (2007) further found that multimedia glosses allow students to understand the main ideas better by making propositional materials easier to access and at the same time, raising students' awareness of the significance of the content. This can explain the reasons why learners in our research also got high scores on the reading comprehension questions that did not require the glossed words directly. Akbulut (2007) also came to the conclusion that the provision of glosses aids students in comprehending the text because it does not interrupt reading process, and it provides a good interaction between the learner and the text, which promotes individualized learning and enhances autonomy. Thus, it allows students to control their own learning process, reading pace, as well as enjoy the interaction that multimedia glosses provide, thereby facilitating reading comprehension.

However, no significant differences were observed in reading comprehension tests among all the conditions. One possible explanation is that reading comprehension is a complicated process, which “involves the smooth interaction between top-down processing (understanding schema, propositions) and bottom-up processing (recognizing or understanding individual vocabulary) and neither alone is sufficient” (Chun and Plass, 1996). In order to promote reading comprehension, besides bottom-up processing which can be facilitated by word-level glossing presentation, top-down processing is also indispensable. Just as Lysenko and Abrami (2014) mentioned, the achievement of reading comprehension includes but is not solely restricted to vocabulary; other factors, such as syntactic complexity, semantic information, and reading strategies also play a vital role in enhancing reading comprehension. Moreover, reading in CALL environment is more challenging for students than reading in the traditional environment since reading in a CALL environment involves hypermedia, which provides flexible information (Ercetin, 2003). Thus, in order to promote reading comprehension, it is of great significance to not only teach students some general reading strategies, including getting the gist of the passage, drawing inferences from the contexts, skimming, and scanning (Hock and Mellard, 2005) but also teach them some useful reading skills that they can use to manage the CALL learning environment, such as how to interpret visual and audio information (Lemke, 1998), when to read the glosses (Venezky, 1994) and so on.

In response to research question 3, from the questionnaire and interview data, it can be concluded that most of the participants hold the opinion that all the glossing modes, particularly L2 definition coupled with visual materials, including pictures and videos, are helpful for their vocabulary learning and reading comprehension. These findings are consistent with the results conducted by AI-Seghayer (2001) and Ramezanali and Faez (2019). Just as AI-Seghayer (2001) mentioned, there are many advantages that videos provide, including better building a mental image, better creating curiosity leading to increased concentration, and combining various modalities (vivid or dynamic image, sound, and printed text). The questionnaire data showed that most of the participants took the view that the inclusion of videos and pictures motivated them to retain vocabulary and read the articles, and the interview data implied that participants considered L2 definition plus visual glossing modes as a motivating practice. These findings support the Cognitive-Affective Theory of Learning with Media. It emphasizes that motivation plays an essential role in multimedia learning and some motivating instructions, for instance, the addition of appealing graphics, tend to foster generative processing (Mayer, 2014).

## Conclusion and Limitations

This study focused on investigating what type of multimedia glossing presentation, including L2 definition only, L2 definition + picture, L2 definition+ audio, and L2 definition + video, better aids EFL learners to comprehend the texts and learn more words. The results supported the positive role of multimedia gloss presentation on vocabulary learning and reading comprehension. They also showed that both visual glossing modes are more effective than audio and L2 definition-only glossing modes on learners' vocabulary acquisition. However, for reading comprehension, all the glossing presentation promotes students' comprehension of the texts and there are no significant differences among the four groups.

The present study has several pedagogical implications for future practice. First, language teachers are strongly encouraged to insert glossing into the computerized texts to promote students' vocabulary acquisition and reading comprehension. During the time of COVID-19, the provision of multimedia glosses not only can help students have more autonomy over their reading but also provide an interesting and effective way to enhance their learning when studying online. Teachers can take the advantage of L2 definition plus video and picture glosses, which can make the learning both interesting and fruitful.

This study by no means poses some limitations. First, the reading comprehension was only measured by multiple-choice questions, so some different tests, such as recall protocols, summary writing, or short answers, may produce different results. Second, other factors, including learners' prior knowledge, proficiency level, may influence the results, so future studies could take these individual differences into consideration. Lastly, due to the time constraints, the study did not measure students' delayed test results, so for future studies, it is suggested to include the delayed test to check the effects on vocabulary retention.

## Data Availability Statement

The raw data supporting the conclusions of this article will be made available by the authors, without undue reservation.

## Ethics Statement

The studies involving human participants were reviewed and approved by College of Information and Business, North University of China. The participants provided their written informed consent to participate in this study.

## Author Contributions

Both authors listed have made a substantial, direct and intellectual contribution to the work, and approved it for publication.

## Conflict of Interest

The authors declare that the research was conducted in the absence of any commercial or financial relationships that could be construed as a potential conflict of interest.

## References

[B1] Abraham L. (2008). Computer-mediated glosses in second language reading comprehension and vocabulary learning: a meta-analysis. Comput. Assist. Lang. Learn. 21, 199–226. 10.1080/09588220802090246

[B2] Abuseileek A. (2011). Hypermedia annotation presentation: the effect of location and type on the EFL learners' achievement in reading comprehension and vocabulary acquisition. Comput. Educ. 57, 1281–1291. 10.1016/j.compedu.2011.01.011

[B3] AI-Seghayer K. (2001). The effect of multimedia annotation modes on L2 vocabulary acquisition: a comparative study. Lang. Learn. Technol. 5, 202–232. 10.125/25117

[B4] Akbulut Y. (2007). Effects of multimedia annotations on incidental vocabulary learning and reading comprehension of advanced learners of English as a foreign language. Instruct. Sci. 35, 499–517. 10.1007/s11251-007-9016-7

[B5] AlRamadhan M. H. (2020). L1 textual glosses and word repetition: facilitative interventions for incidental vocabulary acquisition. Int. J. Instruct. 13, 815–832. 10.29333/iji.2020.13450a

[B6] Andrä C. Mathias B. Schwager A. Macedonia M. von Kriegstein K. (2020). Learning foreign language vocabulary with gestures and pictures enhances vocabulary memory for several months post-learning in eight-year-old school children. Educ. Psychol. Rev. 32, 815–850. 10.1007/s10648-020-09527-z

[B7] Ariew R. Ercetin G. (2004). Exploring the potential of hypermedia annotations for second language reading. Comput. Assist. Lang. Learn. 17, 237–259. 10.1080/0958822042000334253

[B8] Blohm P. (1982). Computer-aided glossing and facilitated learning in prose recall, in New Inquiries in Reading Research and Instruction: Thirty-First Yearbook of the National Reading Conference, eds Niles J. Harris L. (Rochester, NY: National Reading Conference), 24–28.

[B9] Boers F. Warren P. Grimshaw G. Siyanova-Chanturia A. (2017a). On the benefits of multimodal annotations for vocabulary uptake from reading. Comput. Assist. Lang. Learn. 30, 709–725. 10.1080/09588221.2017.1356335

[B10] Boers F. Warren P. He L. Deconinck J. (2017b). Does adding pictures to glosses enhance vocabulary uptake from reading? System 66, 113–129. 10.1016/j.system.2017.03.017

[B11] Bowles M. A. (2004). L2 glossing: To CALL or not to CALL. Hispania 87, 541–552. 10.2307/20063060

[B12] Brandl K. (2002). Integrating Internet-based reading materials into the foreign language curriculum: from teacher- to student-centered approaches. Lang. Learn. Technol. 6, 87–107.

[B13] Chun D. M. Plass J. L. (1996). Effects of multimedia annotations on vocabulary acquisition. Mod. Lang. J. 80, 183–198. 10.1111/j.1540-4781.1996.tb01159.x

[B14] Davis J. N. (1989). Facilitating effects of marginal glosses on foreign language reading. Mod. Lang. J. 73, 41–48. 10.1111/j.1540-4781.1989.tb05308.x

[B15] Ercetin G. (2003). Exploring ESL learners' use of hypermedia reading glosses. CALICO J. 20, 261–283. 10.1558/cj.v20i2.261-283

[B16] González-Fernández B. Schmitt N. (2020). Word knowledge: exploring the relationships and order of acquisition of vocabulary knowledge components. Appl. Linguist. 41, 481–505. 10.1093/applin/amy057

[B17] Hock M. Mellard D. (2005). Reading comprehension strategies for adult literacy outcomes. J. Adolesc. Adult Liter. 49, 192–200. 10.1598/jaal.49.3.3 23087594 PMC3474365

[B18] Kaplan-Rakowski R. Loranc-Paszylk B. (2019). The impact of verbal and nonverbal auditory resources on explicit foreign language vocabulary learning. System 85:102114 10.1016/j.system.2019.102114

[B19] Kim D. Gilman D. A. (2008). Effects of text, audio, and graphic aids in multimedia instruction for vocabulary learning. Educ. Technol. Soc. 11, 114–126.

[B20] Kost C. R. Fost P. Lenzini J. J. (1999). Textual and pictorial glosses: effectiveness of incidental vocabulary growth when reading in a foreign language. Foreign Lang. Ann. 32, 89–113. 10.1111/j.1944-9720.1999.tb02378.x

[B21] Leahy W. Sweller J. (2011). Cognitive load theory, modality of presentation, and the transient information effect. Appl. Cogn. Psychol. 25, 943–951. 10.1002/acp.1787

[B22] Lee H. Lee J. (2015). The effects of electronic glossing types on foreign language vocabulary learning: different types of format and glossary information. Asia Pac. Educ. Res. 24, 591–601. 10.1007/s40299-014-0204-3

[B23] Lemke J. L. (1998). Metamedia literacy: transforming meanings and media, in Handbook of Literacy and Technology: Transformations in a Post-Typographic World, eds Reinking D. McKenna M. C. Labbo L. D. Kieffer R. D. (Hillsdale, NJ: Lawrence Erlbaum), 283–302.

[B24] Lin C. Tseng Y. (2012). Videos and animations for vocabulary learning: a study on difficult words. Turk. Online J. Educ. Technol. 11, 346–355.

[B25] Lomicka L. (1998). To gloss or not to gloss: an investigation of reading comprehension online. Lang. Learn. Technol. 1, 41–50.

[B26] Low R. Sweller J. (2014). The modality principle in multimedia learning, in The Cambridge Handbook of Multimedia Learning, 2nd edn, ed Mayer R. E. (New York, NY: Cambridge University Press), 227–246.

[B27] Lysenko L. Abrami P. (2014). Promoting reading comprehension with the use of technology. Comput. Educ. 75, 162–172. 10.1016/j.compedu.2014.01.010

[B28] Mayer K. M. Yildiz I. B. Macedonia M. von Kriegstein K. (2015). Visual and motor cortices differentially support the translation of foreign language words. Curr. Biol. 25, 530–535. 10.1016/j.cub.2014.11.068 25660537

[B29] Mayer R. (2014). Incorporating motivation into multimedia learning. Learn. Instr. 29, 171–173. 10.1016/j.learninstruc.2013.04.003

[B30] Mayer R. Lee H. Peebles A. (2014). Multimedia learning in a second language: a cognitive load perspective. Appl. Cogn. Psychol. 28, 653–660. 10.1002/acp.3050

[B31] Mayer R. E. (2009). Multimedia Learning, 2nd edn New York, NY: Cambridge University Press.

[B32] Mayer R. E. (2017). Using multimedia for e-learning. J. Comput. Assist. Learn. 33, 403–423. 10.1111/jcal.12197

[B33] Mayer R. E. Moreno R. (2003). Nine ways to reduce cognitive load in multimedia learning. Educ. Psychol. 38, 43–52. 10.1207/S15326985EP3801_6

[B34] Moreno R. (2005). Instructional technology: promise and pitfalls, in Technology-Based Education, eds PytlikZillig L. M. Bodvarsson M. Brunning R. (Greenwich, CT: Information Age Publishing), 1–20.

[B35] Moreno R. Mayer R. E. (2007). Interactive multimodal learning environments: special issue on interactive learning environments: contemporary issues and trends. Educ. Psychol. Rev. 19, 309–326. 10.1007/s10648-007-9047-2

[B36] Morett L. M. (2019). The power of an image: Images, not glosses, enhance learning of concrete L2 words in beginning learners. J. Psycholinguist. Res. 48, 643–664. 10.1007/s10936-018-9623-2 30603870

[B37] Nation I. S. P. (2013). Learning Vocabulary in Another Language, 2nd edn. Cambridge: Cambridge University Press.

[B38] Ouyang J. Huang L. Jiang J. (2020). The effects of glossing on incidental vocabulary learning during second language reading: based on an eye-tracking study. J. Res. Read. 43, 496–515. 10.1111/1467-9817.12326

[B39] Paivio A. (1986). Mental Representations: A Dual Coding Approach. Oxford: Oxford University Press.

[B40] Pintrich P. R. (2003). Motivation and classroom learning, in Handbook of Psychology: Educational Psychology, eds Reynolds W. M. Miller G. E. (New York, NY: Wiley), 103–122.

[B41] Ramezanali N. Faez F. (2019). Vocabulary learning and retention through multimedia glossing. Lang. Learn. Technol. 23, 105–124. 10.125/44685

[B42] Ramezanali N. Uchihara T. Faez F. (2020). Efficacy of multimodal glossing on second language vocabulary learning: a meta-analysis. TESOL Q. 1–23. 10.1002/tesq.579

[B43] Rassaei E. (2018). Computer-mediated textual and audio glosses, perceptual style and L2 vocabulary learning. Lang. Teach. Res. 22, 657–675. 10.1177/1362168817690183

[B44] Repetto C. Pedroli E. Macedonia M. (2017). Enrichment effects of gestures and pictures on abstract words in a second language. Front. Psychol. 8, 21–36. 10.3389/fpsyg.2017.02136 29326617 PMC5736538

[B45] Rott S. (2007). The Effect of frequency of input-enhancements on word learning and text comprehension. Lang. Learn. 57, 165–199. 10.1111/j.1467-9922.2007.00406.x

[B46] Rouhi A. Mohebbi H. (2012). The effect of computer assisted L1 and L2 glosses on L2 vocabulary learning. J. Asia TEFL 9, 1–19.

[B47] Rouhi A. Mohebbi H. (2013). Glosses, spatial intelligence, and L2 vocabulary learning in multimedia context. 3L: Language, Linguistics, Literature®, 19, 75–87.

[B48] Rungwaraphong P. (2020). Using glosses for vocabulary assistance in Thai EFL reading classes: An investigation of preferences, effective types and elements. Electr. J. Foreign Lang. Teach. 17, 301–317.

[B49] Sakar A. Ercetin G. (2005). Effectiveness of hypermedia annotations for foreign language reading. J. Comput. Assist. Learn. 21, 28–38. 10.1111/j.1365-2729.2005.00108.x

[B50] Schmitt N. (2008). Review of review article: instructed second language vocabulary learning. Lang. Teach. Res. 12, 329–363. 10.1177/1362168808089921

[B51] Singh A. Marcus N. Ayres P. (2012). The transient information effect: investigating the impact of segmentation on spoken and written text. Appl. Cogn. Psychol. 26, 848–853. 10.1002/acp.2885

[B52] Sweller J. (1999). Instructional Design in Technical Areas. Camberwell: ACER Press.

[B53] Taylor A. M. (2020). Technology and reading: the effects of CALL glossing. Psychol. Rep. 1–23. 10.1177/0033294120954139 32954975

[B54] Tellier M. (2008). The effect of gestures on second language memorisation by young children. Gesture 8, 219–235. 10.1075/gest.8.2.06tel

[B55] Venezky R. (1994). Literacy and the textbook of the future, in Literacy: A Redefinition, eds Ellsworth N. J. Hedley C. N. Baratta A. N. (Hillsdale, NJ: Lawrence Erlbaum), 39–54.

[B56] Yanagisawa A. Webb S. Uchihara T. (2020). How do different forms of glossing contribute to L2 vocabulary learning from reading? Stud. Second Lang. Acquisit. 42, 411–438. 10.1017/S0272263119000688

[B57] Yeh Y. Wang C. w. (2003). Effects of multimedia vocabulary annotations and learning styles on vocabulary learning. CALICO J. 21, 131 10.1558/cj.v21i1.131-144

[B58] Yoshii M. Flaitz J. (2002). Second language incidental vocabulary retention: the effect of picture and annotation types. CALICO J. 20, 33–58. 10.1558/cj.v20i1.33-58

[B59] Yun J. (2011). The effects of hypertext glosses on L2 vocabulary acquisition: a meta-analysis. Comput. Assist. Lang. Learn. 24, 39–58. 10.1080/09588221.2010.523285

