# Supplementary material for: Multimedia Gloss Presentation: Learners' Preference and the Effects on EFL Vocabulary Learning and Reading Comprehension
Source: Front Psychol. 2021 Feb 4;11:602520. doi: 10.3389/fpsyg.2020.602520 (PMC7891738; doi:10.3389/fpsyg.2020.602520)
Supplement: Supplementary file 1 [file Data_Sheet_1.pdf]

Appendix for reading comprehension questions

Appendix 1. Reading comprehension questions for passage 1

1. What does the author say about the threat of robots?

A) It may constitute a challenge to computer programmers.
B) It accompanies all machinery involving high technology.
C) It can be avoided if human values are translated into their language.
D) It has become an inevitable peril as technology gets more sophisticated.

2. What would we think of a person who invades our personal space according to the author?

A) They are aggressive.
B) They are outgoing.
C) They are ignorant.
D) They are ill-bred.

3. How do robots learn human values?

A) By interacting with humans in everyday life situations.
B) By following the daily routines of civilized human beings.
C) By picking up patterns from massive data on human behavior.
D) By imitating the behavior of property brought-up human beings.

4. What will a well-programmed robot do when facing an unusual situation?

A) keep a distance from possible dangers.
B) Stop to seek advice from a human being.
C) Trigger its built-in alarm system at once.
D) Do sufficient testing before taking action.

5. What is most difficult to do when we turn human values into a programmable code?

A) Determine what is moral and ethical.
B) Design some large-scale experiments.

C) Set rules for man-machine interaction.

D) Develop a more sophisticated program.

Appendix 2. Reading comprehension questions for passage 2

1. The aim of the study in the Journal of the American Geriatrics Society is____.

A) to see whether people's personality affects their life span

B) to find out if one's lifestyle has any effect on their health

C) to investigate the role of exercise in living a long life

D) to examine all the factors contributing to longevity

2. What does the author imply about outgoing and sympathetic people?

A) They have a good understanding of evolution.

B) They are better at negotiating an agreement.

C) They generally appear more resourceful.

D) They are more likely to get over hardship.

3. What finding of the study might prove somewhat out of our expectation?

A) Easy-going people can also live a relatively long life.

B) Personality characteristics that prove advantageous actually vary with times.

C) Such personality characteristics as self-discipline have no effect on longevity.

D) Readiness to accept new ideas helps one enjoy longevity.

4. What does the recent study of Norwegian mothers show?

A) Children's personality characteristics are invariably determined by their mothers.

B) People with unhealthy eating habits are likely to die sooner.

C) Mothers' influence on children may last longer than fathers'.

D) Mothers' negative personality characteristics may affect their children's life spans.

5. What can we learn from the findings of the two new studies?

A) Anxiety and depression more often than not cut short one's life span.

B) Longevity results from a combination of mental and physical health.

C) Personality plays a decisive role in how healthy one is.

D) Health is in large part related to one's lifestyle.

Appendix 3. Reading comprehension questions for passage 3

1. What does the author mainly discuss in the passage?

A) The reasons behind the plunge of oil prices.

B) Possible ways to stimulate the global economy.

C) The impact of cheap oil on global economic growth.

D) The effect of falling oil prices on consumer spending.

2. Why do some experts believe cheap oil will stimulate the global economy?

A) Manufacturers can produce consumer goods at a much lower cost.

B) Lower oil prices have always given a big boost to the global economy.

C) Oil prices may rise or fall but economic laws are not subject to change.

D) Consumers will spend their saving from cheap oil on other commodities.

3. What happens in many oil-exporting countries when oil prices go down?

A) They suspend import of necessities from overseas.

B) They reduce production drastically to boost oil prices.

C) They use their money reserves to back up consumption.

D) They try to stop their economy from going into free-fall.

4. How does Carl Weinberg view the current oil price plunge?

A) It is one that has seen no parallel in economic history.

B) Its negative effects more than cancel out its positive effects.

C) It still has a chance to give rise to a boom in the global economy.

D) Its effects on the global economy go against existing economic laws.

5. Why haven't falling oil prices boosted the global economy as they did before?

A) People are not spending all the money they save on gas.
B) The global economy is likely to undergo another recession.
C) Oil importers account for a larger portion of the global economy.
D) People the world over are afraid of a further plunge in oil prices.

Appendix 4. Reading comprehension questions for passage 4

1. According to one study, what do green spaces do to people?

A) Improve their work efficiency.
B) Add to their sustained happiness.
C) Help them build a positive attitude towards life.
D) Lessen their concerns about material well-being.

2. What does Dr. White say people usually do to make themselves happier?

A) Earn more money.
B) Settle in an urban area.
C) Gain fame and popularity.
D) Live in a green environment.

3. What does Dr. White try to find out about living in a greener urban area?

A) How it affects different people.
B) How strong its positive effect is.
C) How long its positive effect lasts.
D) How it benefits people physically.

4. What did Dr. White's research reveal about people living in a green environment?

A) Their stress was more apparent than real.
B) Their decisions required less deliberation.
C) Their memories were greatly strengthened.
D) Their communication with others improved.

5. According to Dr. White, what should the government do to build more green spaces in cities?

A) Find financial support.

B) Improve urban planning.

C) Involve local residents in the effort.

D) Raise public awareness of the issue.

Appendix 5. Reading comprehension questions for passage 5

1. What do we learn about McDonald's inclusion of toys in its Happy Meals?

A) It may shed light on people's desire to crack a secret.

B) It has proved to be key to McDonald's business success.

C) It appeals to kid's curiosity to find out what is hidden inside.

D) It may be a pleasant way for kids to reduce their food intake.

2. What is the finding of the researchers led by Martin Reimann?

A) Reducing food intake is not that difficult if people go to McDonald's more.

B) Most kids and adults don't actually feel hungry when they eat half of their meal.

C) Eating a smaller portion of food does good to the health of kids and adults alike.

D) Most kids and adults would choose a smaller meal that came with a non-food item.

3. What is most interesting in Martin Reimann's finding?

A) Kids preferred an award in the form of money to one in the form of a toy.

B) Adults chose the smaller portion on the mere promise of a future award.

C) Both kids and adults felt satisfied with only half of their meal portions.

D) Neither children nor adults could resist the temptation of a free toy.

4. How does Martin Reimann interpret his finding?

A) The emotional component of the prizes is at work.

B) People now care more about quality than quantity.

C) People prefer certainty awards to possible awards.

D) The desire for a future reward is overwhelming.

5. What can we infer from Martin Reimann's finding?

A) People should eat much less if they wish to stay healthy and happy.
B) More fast-food restaurants are likely to follow McDonald's example.
C) We can lead people to eat less while helping the restaurant business.
D) More studies are needed to find out the impact of emotion on behavior.

Appendix 6. Reading comprehension questions for passage 6

1. According to the new report, real estate development in 2015 will witness ______.

A) an accelerating speed
B) a shift to city centers
C) a new focus on small cities
D) an ever-increasing demand

2. What characterizes "24-h cities" like New York?

A) People can live without private cars.
B) People are generally more competitive.
C) People can enjoy services around the clock.
D) People are in harmony with the environment.

3. Why are Millennials reluctant to buy a house?

A) They can only afford small apartments.
B) The house prices are currently too high.
C) Their parents' bad experience still haunts them.
D) They feel attached to the suburban environment.

4. What might hinder real estate development in the USA?

A) The continuing economic recession in the country.
B) The lack of confidence on the part of investors.
C) The fierce global competition.
D) The worsening infrastructure.

5. How do most of the respondents in the survey feel about the US real-estate market in 2015?

A) Pessimistic.
B) Hopeful.
C) Cautious.
D) Uncertain.

Appendix 7. Reading comprehension questions for passage 7

1. What have past behavioral studies found about our brain?

A) Its capacity actually knows no limits.
B) It grows sophisticated with practice.
C) It keeps our most precious memories until life's end.
D) New information learned pushes old information out.

2. What is the benefit of forgetting?

A) It frees us from painful memories.
B) It helps slow down our aging process.
C) It facilitates our access to relevant information.
D) It prevents old information from forming associations.

3. What is the emphasis of current studies of memory?

A) When people tend to forget.
B) What contributes to forgetting.
C) How new technology hinders memory capacity.
D) Why learning and forgetting are complementary.

4. What do people find about their rare ability to remember every detail of their life?

A) It adds to the burden of their memory.
B) It makes their life more complicated.
C) It contributes to their success in life.
D) It constitutes a rare object of envy.

5. What docs the passage say about forgetting?

A) It can enlarge our brain capacity.
B) It helps get rid of negative memories.
C) It is a way of organizing our memories.
D) It should not cause any alarm in any way.

Appendix 8. Reading comprehension questions for passage 8

1. What would be the impact of the extensive use of driverless cars?

A) People would be driving in a more civilized way.
B) It would save local governments a lot of money.
C) More policemen would be patrolling the streets.
D) Traffic regulations would be a thing of the past.

2. How would the elderly and the disabled benefit from driverless cars?

A) They could enjoy greater mobility.
B) They would suffer no road accidents.
C) They would have no trouble driving.
D) They could go anywhere they want.

3. What would be the negative impact of driverless cars?

A) The conflict between labor and management intensify.
B) The gap between various sectors of society would be widened.
C) Professional drivers would have a hard time adapting to new road conditions.
D) Numerous professional drivers would have to find new ways of earning a living.

4. What is the result of the introduction of new technologies in energy industries?

A) Political dissatisfaction.
B) Retraining of employees.
C) Fossil fuel conservation.
D) Business restructuring.

5. What does the author suggest businesses and the government do?

A) Keep pace with technological developments.

B) Make new technologies affordable to everyone.

C) Enable everyone to benefit from new technologies.

D) Popularize the use of new technologies and devices.
